# Dysregulation of *NIPBL* leads to impaired *RUNX1* expression and haematopoietic defects

**DOI:** 10.1111/jcmm.15269

**Published:** 2020-04-23

**Authors:** Mara Mazzola, Alex Pezzotta, Grazia Fazio, Alessandra Rigamonti, Erica Bresciani, Germano Gaudenzi, Maria Chiara Pelleri, Claudia Saitta, Luca Ferrari, Matteo Parma, Monica Fumagalli, Andrea Biondi, Giovanni Cazzaniga, Anna Marozzi, Anna Pistocchi

**Affiliations:** ^1^ Dipartimento di Biotecnologie Mediche e Medicina Traslazionale Università degli Studi di Milano Milano Italy; ^2^ Centro Ricerca Tettamanti, Fondazione Tettamanti, Università degli Studi di Milano-Bicocca Monza Italy; ^3^ Oncogenesis and Development Section National Human Genome Research Institute, National Institutes of Health Bethesda MD USA; ^4^ Laboratorio Sperimentale di Ricerche di Neuroendocrinologia Geriatrica e Oncologica Istituto Auxologico Italiano, IRCCS Cusano Milanino Italy; ^5^ Department of Experimental, Diagnostic and Specialty Medicine (DIMES) Unit of Histology, Embryology and Applied Biology University of Bologna Bologna Italy; ^6^ Dipartimento di Scienze Cliniche e Comunità Università degli Studi di Milano Milano Italy; ^7^ Clinica Ematologica e Centro Trapianti di Midollo Osseo, Ospedale San Gerardo Università di Milano‐Bicocca Monza Italy

**Keywords:** AML, haematopoiesis, NIPBL, RUNX1, zebrafish

## Abstract

The transcription factor *RUNX1,* a pivotal regulator of HSCs and haematopoiesis, is a frequent target of chromosomal translocations, point mutations or altered gene/protein dosage. These modifications lead or contribute to the development of myelodysplasia, leukaemia or platelet disorders. A better understanding of how regulatory elements contribute to fine‐tune the *RUNX1* expression in haematopoietic tissues could improve our knowledge of the mechanisms responsible for normal haematopoiesis and malignancy insurgence. The cohesin RAD21 was reported to be a regulator of *RUNX1* expression in the human myeloid HL60 cell line and during primitive haematopoiesis in zebrafish. In our study, we demonstrate that another cohesin, NIPBL, exerts positive regulation of *RUNX1* in three different contexts in which *RUNX1* displays important functions: in megakaryocytes derived from healthy donors, in bone marrow samples obtained from adult patients with acute myeloid leukaemia and during zebrafish haematopoiesis. In this model, we demonstrate that alterations in the zebrafish orthologue *nipblb* reduce *runx1* expression with consequent defects in its erythroid and myeloid targets such as *gata1a* and *spi1b* in an opposite way to rad21. Thus, also in the absence of *RUNX1* translocation or mutations, additional factors such as defects in the expression of *NIPBL* might induce haematological diseases.

## 1 INTRODUCTION

In all vertebrates, the RUNX family of transcriptional regulators containing the runt domain (RD) comprises three isoforms: RUNX1, RUNX2 and RUNX3 that, together with the non‐DNA‐binding CBFβ subunit, regulate many developmental processes.^1^, ^2^ The RUNX members specify their functions depending on their cellular and tissue expression: RUNX1 plays a key role in blood development, primarily in the haematopoietic stem cells (HSCs), RUNX2 is manly involved in bone morphogenesis, and RUNX3 in cell growth of neurons, epithelial cells and T cells. However, the three RUNX proteins could exert biological activities also in other organs^3^, ^4^, ^5^; for example, RUNX2 and RUNX3 are known to play a role during haematopoiesis together with RUNX1. In addition, all the *RUNX* genes are transcribed by a distal and a proximal promoter (*P1* and *P2,* respectively) in two main isoforms that differ in the 5′UTR and in the coding sequence of the first exon.^6^, ^7^ The *P1* and *P2RUNX* transcripts are differentially expressed in diverse cell types and during specific developmental stages. Indeed, *P1* and *P2RUNX1* promoters have been reported to have specific activity patterns in the different haematopoietic lineages during development.^8^

RUNX1 function during haematopoiesis is strictly regulated by post‐transcriptional and post‐translational modifications such as alternative splicing, acetylation, methylation, phosphorylation and ubiquitination.^9^ As transcription factor, RUNX1 targets multiple genes, many of which are also pivotal transcriptional regulators involved in the formation of all haematopoietic lineages including the haematopoietic‐specific member of E‐twenty‐six (ETS) family, PU.1.^10^, ^11^ Furthermore, the activity of RUNX1 is carried out by its interaction with different proteins fundamental during haematopoiesis such as GATA1, PU.1, CEBPA, PAX5 and ETS1.^10^, ^12^, ^13^, ^14^

Given the high complexity in RUNX1 expression and function, its deregulation is commonly associated with haematopoietic diseases. Depletion of *Runx1* in mice and zebrafish models leads to severe defects or complete absence of definitive haematopoiesis.^15^, ^16^, ^17^, ^18^ *RUNX1* is frequently involved in chromosomal translocations observed in acute leukaemias, such as ETV6‐RUNX1 in t(12;21) and RUNX1‐EVI1 in t(3;21),^19^ while the formation of the chimeric protein RUNX1‐CBF2T1 (AML1‐ETO) is associated with the M2 subtype of acute myeloid leukaemia (AML).^20^, ^21^ *RUNX1* mutations determine the familial platelet disorder with a propensity for AML (AML/FPD) and the minimally differentiated acute myeloid leukaemia (AML/M0).^22^ Importantly, regulation of *RUNX1* dosage is essential for the maintenance of normal haematopoiesis^23^ and several haematopoietic transcription factors are deputed to regulate *RUNX1* expression such as Gata2, Ets factors (Fli‐1, Elf‐1 and Pu.1) and the SCL/Lmo2/Ldb1 complex.^24^ In zebrafish, the subunit Rad21 of the cohesin complex has been identified as a regulator of *runx1* through a forward genetic screen,^25^ and multiple predicted and in vivo validated binding sites of Rad21 have been shown to be involved in the regulation of the zebrafish *runx1*.^26^

In this work, we demonstrate that NIPBL, another member of the cohesin complex, positively regulates *RUNX1* expression in two different contexts in which it exerts important functions: normal cord blood megakaryocytes derived from healthy donors and bone marrow samples derived from adult AML patients. In addition, we generate a zebrafish model in which the *nipblb*‐mediated dysregulation of *runx1* expression leads to haematopoietic defects resulting in decreased expression of the erythroid marker *gata1a* and reduction of mature circulating erythrocytes, and increased expression of myeloid precursors positive for the *spi1b* marker. Our data confirm the regulatory loop between RUNX1‐GATA1 and PU.1 during haematopoiesis and highlight a new role of NIPBL on top of this route.

## 2 MATERIALS AND METHODS

### 2.1 Patients

Diagnostic bone marrow samples from 34 adult patients affected by AML were collected and characterized for specific molecular aberrancies, including translocations t(9;22), t(8;21) and inv(16), in accordance with specific clinical protocol requirements. The analysed patients belong to different French‐American‐British (FAB) classification systems (FABs), excluding M3; therefore, all patients were negative for translocation t(15;17) (Table 1). Bone marrow of healthy individuals was collected as controls for gene expression assays, upon appropriate informed consent ASG‐A‐052A approved on 8 May 2012 by Azienda Socio‐Sanitaria of Monza (ASST‐Monza). Human material and derived data were used in accordance with the Declaration of Helsinki.

**TABLE 1:** Clinical Features of patients' cohort

Age at onset		Karyotype	FAB classification	NPM	FLT3‐ITD	t(9;22)	t(8;21)	inv(16)
1	47	46,XX,t(10;11)(p11;p15)[20]	M0	NEG	NEG	NEG	NEG	NEG
2	49	46,XY[20]	M0/M1	NEG	NEG	NEG	NEG	NEG
3	48	46,XX[20]	M1	NEG	NEG	NEG	NEG	NEG
4	72	47,XY,+mar[10]/46,XY[10]	M2	NEG	NEG	NEG	NEG	NEG
5	58	46,XX,t(3;5)(q25;q34)[20]	M2	NEG	NEG	NEG	NEG	NEG
6	59	46,XY[20]		NEG	POS	NEG	NEG	NEG
7	33	46,XY[15]	M1	NEG	POS	NEG	NEG	NEG
8	30	46,XY[20]	M5	NEG	POS	nk	NEG	NEG
9	58	46,XY,inv(16)(p13q22)[20]	M4	NEG	POS	nk	NEG	POS
10	76	nk	M5	NEG	POS	nk	NEG	NEG
11	78	46,XX[27]	M4	NEG	POS	nk	NEG	NEG
12	53	46,XY[22]	M4	NEG	POS	nk	NEG	NEG
13	64	46,XX[20]	M5	NEG	POS	nk	NEG	NEG
14	75	46,XY[26]	M4	NEG	POS	nk	NEG	NEG
15	39	46,XY[20]	M1	POS (A)	NEG	NEG	NEG	NEG
16	47	46,XX[20]	M5	POS (A)	NEG	NEG	NEG	NEG
17	63	46,XY,t(8;14)(q24;q32),add(13q34)[18]/46,XY[9]	nk	POS (D)	NEG	nk	NEG	NEG
18	58	46,XY/47,XY,+8[7/10]	nk	POS (QM)	NEG	nk	NEG	NEG
19	50	46,XX[20]	M4	POS (A)	NEG	nk	NEG	NEG
20	77	46,XY[20]	nk	POS (A)	NEG	nk	NEG	NEG
21	54	46,XX,t(9;22)(q34;q11)[14]/46,XX[6]	M4	POS (A)	NEG	POS	NEG	NEG
22	60	46,XX[6]	nk	POS	NEG	nk	NEG	NEG
23	62	46,XX[25]	M5	POS (A)	NEG ITD/POS D835/D836	nk	NEG	NEG
24	58	46,XX[20]	nk	POS (A)	NEG	nk	NEG	NEG
25	48	46,XX[20]	M4	POS (A)	POS	NEG	NEG	NEG
26	51	46,XX[20]	M5	POS (A)	POS	NEG	NEG	NEG
27	68	46,XX[20]	M4	POS (A)	POS ITD/POS D835/D836	NEG	NEG	NEG
28	46	46,XY[20]	M2	POS	POS	NEG	NEG	NEG
29	39	46,XX[22]	M1	POS (A)	POS	nk	NEG	NEG
30	58	46,XY	M5	POS (A)	POS	nk	NEG	NEG
31	35	46,XY,?r(18)(?)[16]/47,idem,+8[3]/46,XY[1]	nk	POS (B)	POS	nk	NEG	NEG
32	58	46,XY[24]	M1	POS (A)	POS	nk	NEG	NEG
33	70	46,XY[20]	M5	POS (A)	POS	nk	NEG	NEG
34	12	46,XY[24]	nk	POS (A)	POS	NEG	NEG	NEG

### 2.2 Animals

Zebrafish embryos were raised and maintained according to international (European Union Directive 2010/63/EU) and national (Italian decree no. 26 of 4 March 2014) guidelines on the protection of animals used for scientific purposes. The fish were maintained under standard conditions in the fish facilities of Bioscience Dept, University of Milan, Via Celoria 26‐20133 Milan, Italy (Aut. Prot, n. 295/2012‐A—20 December 2012). We express the embryonic ages in hours post‐fertilization (hpf) and days post‐fertilization (dpf). Zebrafish AB strains obtained from the Wilson laboratory (University College London, London, UK) and *Tg(fli1a:EGFP)^y1^* ^27^ were maintained at 28°C on a 14‐h light/10‐h dark cycle. Embryos were collected by natural spawning, staged according to Ref. 28 and raised at 28°C in fish water (Instant Ocean, 0,1% Methylene Blue) in Petri dishes, according to established techniques. To prevent pigmentation, 0,003% 1‐phenyl‐2‐thiourea (PTU, Sigma‐Aldrich) was added to the fish water prior to 24 hpf. Before observations and picture acquisitions, embryos were washed, dechorionated and anaesthetized, with 0.016% tricaine (ethyl 3‐aminobenzoate methanesulfonate salt; Sigma‐Aldrich).

### 2.3 Reverse transcription and real‐time quantitative polymerase chain reaction assays (RT‐qPCR)

RNA was extracted from human and zebrafish embryos using TRIzol reagents (Life Technologies), following the manufacturer's protocol. For human samples and RT‐qPCR experiments, Superscript II enzyme (Life Technologies) was used for cDNA synthesis. For this set of experiments, a LightCycler 480II (Roche Diagnostics, Basel, Swiss) was used. Probes were selected according to the Software Probe Finder (Roche Diagnostics) and are reported in Table 2. *hGUS* gene was used as reference gene in human patients and cells derived from healthy donors as standard control. For zebrafish samples, DNase I RNase‐free (Roche Diagnostics) treatment was performed to avoid possible genomic contamination and 1 μg of RNA was reverse‐transcribed using the “ImProm‐II™ Reverse Transcription System” (Promega). RT‐qPCRs were carried out in a total volume of 20 μl containing 1X iQ SYBR Green Supermix (Promega), using proper amount of the RT reaction and a mixture of oligo(dT) and random primers according to manufacturer's instructions. RT‐qPCRs were performed using the Bio‐Rad iCycler iQ Real‐Time Detection System (Bio‐Rad). For normalization purposes, *rpl8* expression levels were tested in parallel with the gene of interest. Primers are reported in Table 3. Expression levels in the Y‐axis were relative to the control.

**TABLE 2:** Human primer sequences and probe numbers used in qPCR experiments

PRIMER	length	sequence	PROBE
hGUS‐L	20	CGCCCTGCCTATCTGTATTC	57
hGUS‐R	20	TCCCCACAGGGAGTGTGTAG	
hNIPBL‐L	19	CTATGCGAACAGCCCAAAA	55
hNIPBL‐R	24	TTCACCTTGCTTACTACCACATTT	
hRAD21‐L	20	ATTGACCCAGAGCCTGTGAT	62
hRAD21‐R	20	GGGGAAGCTCTACAGGTGGT	
HRUNX1‐L	18	ACAAACCCACCGCAAGTC	21
HRUNX1‐R	23	CATCTAGTTTCTGCCGATGTCTT	
HSPI1‐L	20	CTGGAGTTCCCCAATCACAT	25
HSPI1‐R	23	TGATTTCAGACATGACAAAAGGA	

**TABLE 3:** Zebrafish primer sequences used in qPCR experiments

PRIMER	Length	Sequence
zrpl8‐L	21	CTCCGTCTTCAAAGACCATGT
zrpl8‐R	21	TCCTTCACGATCCCCTTGATG
zP1‐runx1‐L	20	ATGGCCTCCAACAGCATCTT
zP2‐runx1‐L	20	GAGCCGAAACTCACGGAGAC
zrunx1 common‐R	20	GCAAACCCTCGCTCATCTTC
zspi1b‐L	19	GCCATTTCATGGACCCAGG
zspi1b‐R	19	ACACCGATGTCCGGGGCAA
zgata1a‐L	26	AACGACATCTTCAATACTACACTTGC
zgata1a‐R	18	GGACACCCAACGAGAAGG

### 2.4 In situ hybridization, *o*‐dianisidine and immunofluorescence analyses

Whole‐mount in situ hybridization (WISH) experiments were carried out as described by Thisse et al.^29^ For quantification of the observed phenotypes, WISH experiments were done at least in 3 independent batches of embryos (minimum 15‐20 embryos for each category). Embryos were fixed overnight in 4% paraformaldehyde (PFA, Sigma‐Aldrich) in phosphate‐buffered saline (PBS) at 4°C, and then dehydrated stepwise to methanol and stored at −20°C. Antisense riboprobes were previously in vitro labelled with modified nucleotides (*i.e.* digoxigenin, Roche Diagnostics). *runx1*,^30^ *spi1b* ^31^ and *gata1a* ^32^ probes were synthesized according to literature. To detect haemoglobin activity, *o*‐dianisidine (Sigma) staining was performed as described in Ref. 33. Controls and MO‐injected embryos at the same developmental stage were scored from 1 to 3 according to the intensity of the staining by microscopy, and *o*‐dianisidine‐positive cells on the yolk surface and in the Caudal haematopoietic tissue (CHT) were compared.

### 2.5 Injections

Injections were carried out on one‐ to two‐cell stage embryos. Details of concentration and sequence of *nipblb* morpholino (*nipblb*‐MO, Gene Tools, Oregon, US) and *rad21*‐MO (Gene Tools) are described in Ref. ^34^ and Ref. ^25^, respectively. In all experiments, MO‐injected embryos were compared to embryos at the same developmental stage injected with the same amount of a ctrl‐MO that has no target in zebrafish (Gene Tools LLC). The *runx1/*PCS2+ construct was kindly provided by C.E. Burns^18^ and injected at a concentration of 200 pg/embryo.

### 2.6 Statistical analyses

For RT‐qPCR experiments, data were statistically analysed applying one‐way analysis of variance (ANOVA), defining *P* ≤ .05 (*), *P* ≤ .01 (**) and *P* ≤ .001 (***) as statistically significant values.^35^ Data were analysed using the comparative ΔΔCt method. Both ANOVA and standard deviation (SD) values refer to data from triplicate samples. In zebrafish, at least three different experiments were done for each analysis.

The degree of linear relationship between *RAD21*, *NIPBL*, *RUNX1, MPL* and *SPI1* expression levels was calculated using Spearman's correlation coefficient (r value).

### 2.7 TRAM analysis

TRAM (Transcriptome Mapper) software^36^ allows the import, decoding of probe set identifiers to gene symbols via UniGene data parsing,^37^ integration and normalization of gene expression data in tab‐delimited text format for the generation and analysis of transcriptome maps. We analysed the transcriptome map previously obtained from a gene expression profile datasets for normal human megakaryocytes (MK) cells derived from healthy donors.^38^ The dataset is composed of 19 samples previously described (Pool D in Ref. ^38^). In particular, we used the function "Export" of TRAM software in order to obtain normalized expression values assigned to *NIPBL*, *RAD21*, *RUNX1* and *MPL* genes for each sample. The degree of linear relationship between *RAD21*, *NIPBL*, *RUNX1*, *MPL* and *SPI1* expression levels was calculated using Spearman's correlation coefficient (r value).

## 3 RESULTS

### 3.1 Positive correlation between *NIPBL* and *RUNX1* expression in normal megakaryocytes derived from healthy donors and bone marrow cells derived from adult AML patients

**FIGURE 1:**
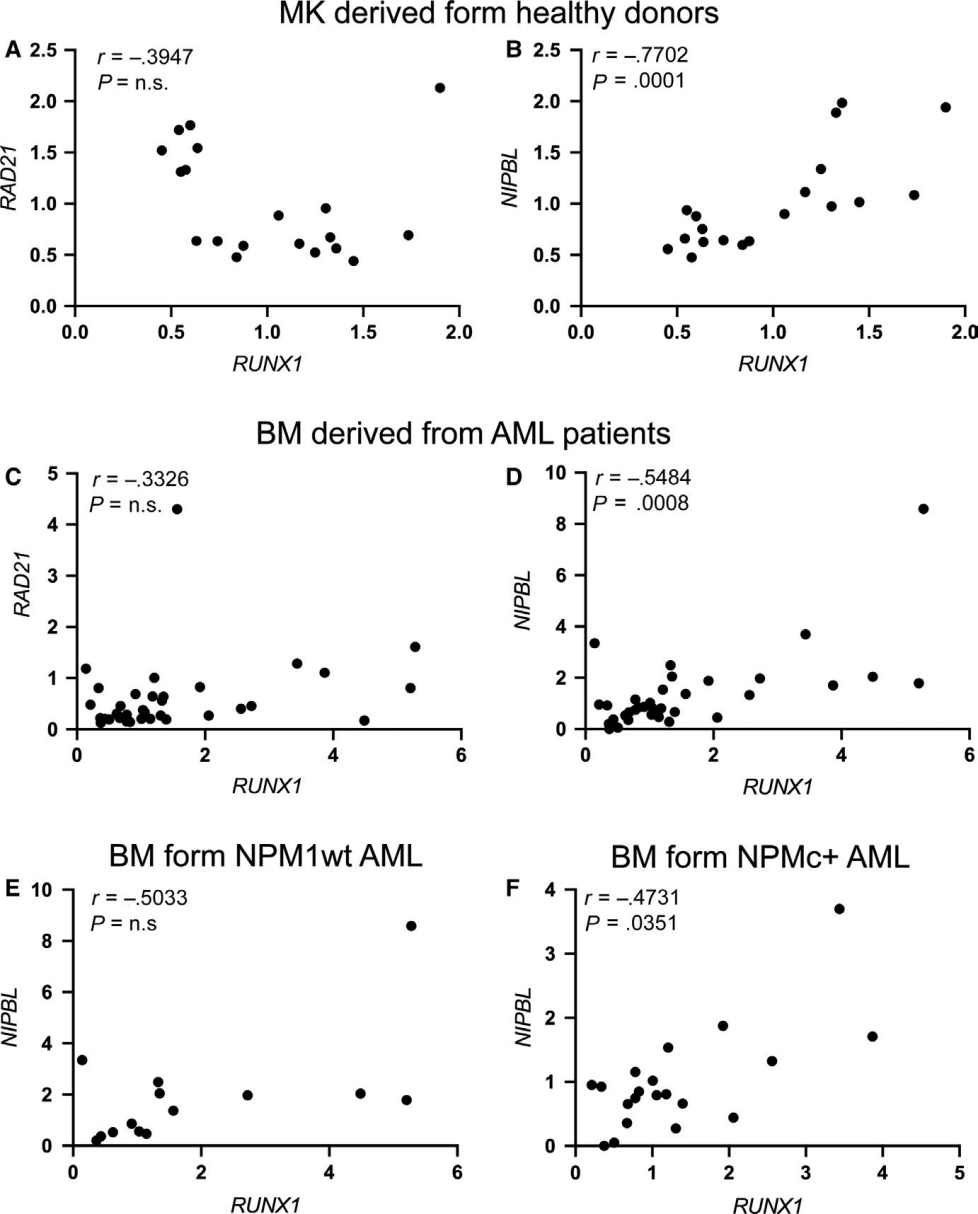
Positive correlation between *NIPBL* and *RUNX1* expression in megakaryocytes derived from healthy donors and in bone marrow cells derived from 34 adult AML patients. A and B, Spearman's correlation between *RUNX1* and *RAD21* (A) or *NIPBL* (B) in cord blood megakaryocytes (MK) derived from healthy donors. C and D, Spearman's correlation between *RUNX1* and *RAD21* (C) or *NIPBL* (D) in bone marrow cells (BM) derived from 34 adult AML patients without aberrant *RUNX1* alterations (mutations or translocations). E and F, Spearman's correlation between *RUNX1* and *NIPBL* in 34 adult AML patients without (NPM1wt) (E) or with NPM1 mutation (NPMc+) (F). Spearman's correlation analysis showed a significant positive correlation of the ratio of *RUNX1* expression only versus *NIPBL*, not versus *RAD21,*. *r* = Spearman's correlation coefficient

*RUNX1* expression has been reported to be regulated by the cohesin subunit RAD21 and the CTCF insulator in human myelocytic leukaemia cells HL‐60.^26^ As RUNX1 is pivotal in the differentiation of megakaryocytes and myeloid lineages, we investigated the relative expression of *RAD21* and *RUNX1* in two different contexts in which *RUNX1* exerts important functions: the differentiation of the megakaryocytes and myeloid compartments under physiological and pathological conditions. For the megakaryocytes compartment in physiological condition, we performed in silico analyses of quantitative transcriptome maps, using TRAM (Transcriptome Mapper) software, which allows import and effective integration of data obtained by different experimenters, experimental platforms and data sources.^36^ In megakaryocytes (MK) derived from healthy donors, *RAD21* expression did not correlate with the expression levels of *RUNX1* (Figure 1A)*.* Conversely, we found a positive correlation between the expression of *RUNX1* and that of *NIPBL*, another member of the cohesin complex (Figure 1B). To explore the myeloid compartment under pathological condition, we used bone marrow (BM) cells derived from adult AML patients. Similar to TRAM analyses, when *RAD21* and *RUNX1* expressions were investigated in a cohort of 34 AML adult patients without anomalies in chromosome 21 that contains the *RUNX1* locus, no significant correlation was reported (Figure 1C). Conversely, we observed the positive *NIPBL/RUNX1* correlation already detected in megakaryocytes (Figure 1D).

We previously showed that *NIPBL* transcript abundance is decreased in AML patients carrying the mutated *NUCLEOPHOSMIN1 (NPM1)*, which transfers NPM1 in the cytoplasm (NPMc+), compared to the NPM1 wild‐type (NPM1wt).^34^ Therefore, we analysed the correlation between the expression of *NIPBL* and *RUNX1* in BM cells derived from 20 patients NPMc+, selected among the 34 AML patients, compared to 14 patients NPM1wt and found a significant positive correlation in NPMc+ but not in NPM1wt AML patients (Figure 1E‐F). Taken together, these findings suggest a new role for NIPBL, different from that of RAD21, in the regulation of *RUNX1* expression and that aberrant expression of *NIPBL,* such as in AML patients with NPM+ mutation, might lead to alteration in *RUNX1* transcript levels.

### 3.2 Knock‐down of *nipblb* specifically reduces *runx1* expression in zebrafish

**FIGURE 2:**
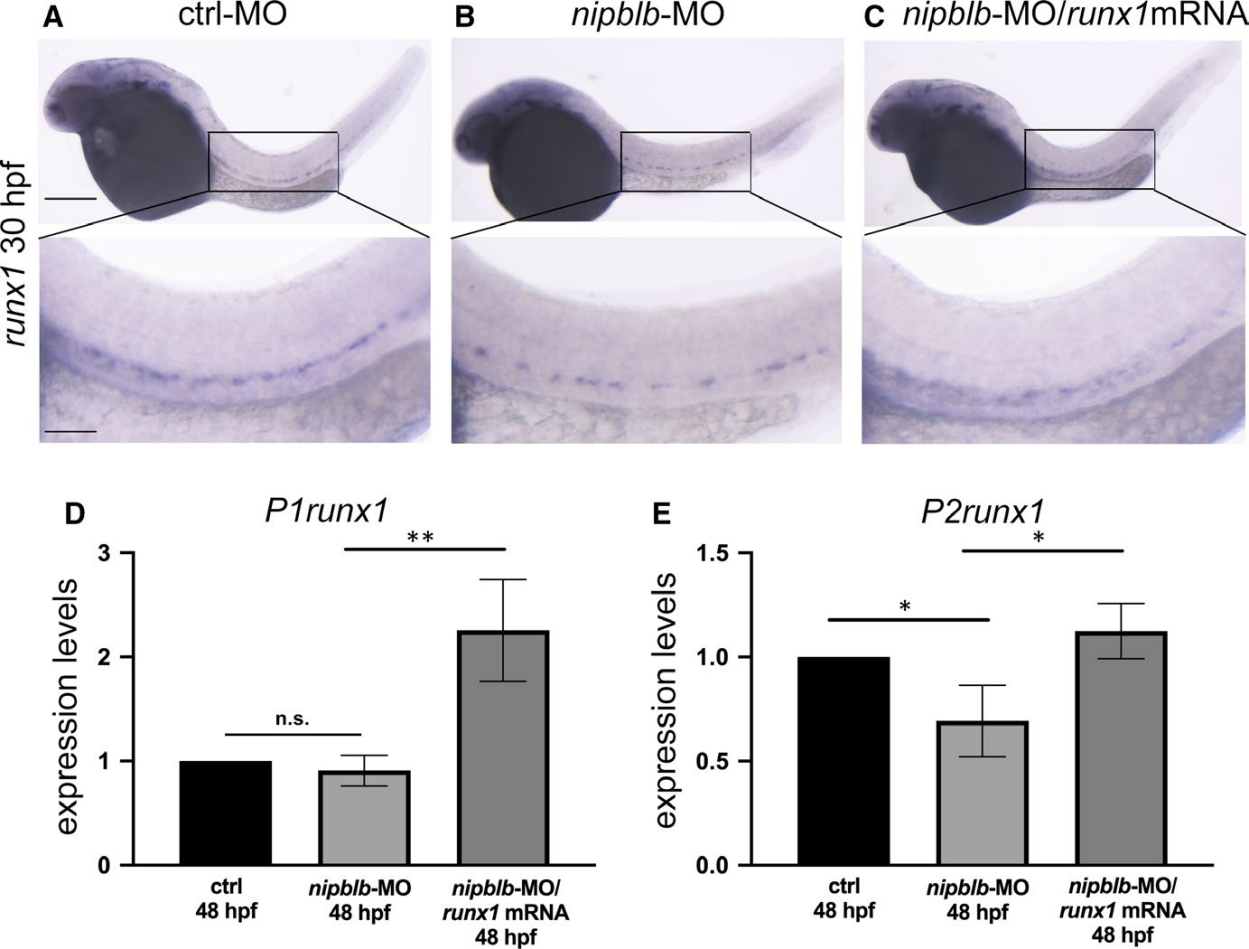
*runx1* expression is specifically reduced following *nipblb* down‐regulation. A‐C WISH analyses of *runx1* expression at the stage of 30 hpf in embryos injected with control morpholino (ctrl‐MO*)* (A), *nipblb*‐MO (B) and *nipblb*‐MO with *runx1*‐mRNA (C)*.* The *runx1* expression in the caudal region (higher magnification in the box) is reduced following *nipblb* down‐regulation and rescued in embryos co‐injected with *nipblb*‐MO and *runx1* mRNA. E‐F, RT‐qPCR analyses of the *P1runx1* (D) and *P2runx1* (E) isoforms in ctrl‐MO‐, *nipblb*‐MO‐ and *nipblb*‐MO/*runx1*mRNA‐injected embryos at 48 hpf. Scale bars indicate 100 μm. One‐way ANOVA with Bonferroni correction, ^**^ *P* < .01, ^*^ *P* < .05, n.s: non‐significant

To confirm the positive correlation between *NIPBL* and *RUNX1* observed in human, we took advantage of a zebrafish model with down‐regulation of *nipblb*, the orthologue of the human *NIPBL*, previously generated in our laboratory.^34^ The expression of *runx1* was analysed in embryos at 30 and 48 hpf as definitive HSCs arise from the vascular endothelium from these developmental stages. Moreover, we verified that both *P1*‐*P2runx1* isoforms were highly expressed from 24 hpf (Figure S1). WISH analyses showed a reduction of the *runx1* transcript in the aorta‐gonad mesonephric (AGM) tissue in *nipblb*‐MO‐injected embryos compared to controls at the same developmental stage. The injection of the full‐length *runx1*/mRNA rescued this phenotype as expected (Figure 2A‐C). As the full‐length *runx1* riboprobe does not distinguish between the *P1*‐ and *P2runx1* isoforms present in zebrafish,^8^ we performed RT‐qPCR analysis of both isoforms, revealing a significant reduction exclusively in *P2runx1* transcript levels following *nipblb* down‐regulation*.* The expression of both isoforms was increased in embryos injected with *nipblb*‐MO and *runx1*mRNA, confirming the efficacy of the *runx1* overexpression (Figure 2D‐E). These results provide evidence that *nipblb* knock‐down causes the reduction of *runx1* in zebrafish, confirming the positive correlation between *NIPBL* and *RUNX1* expression observed in normal megakaryocytes and in BM of  AML patients.

### 3.3 NIPBL‐mediated RUNX1 down‐regulation impairs the expression of RUNX1 target genes

**FIGURE 3:**
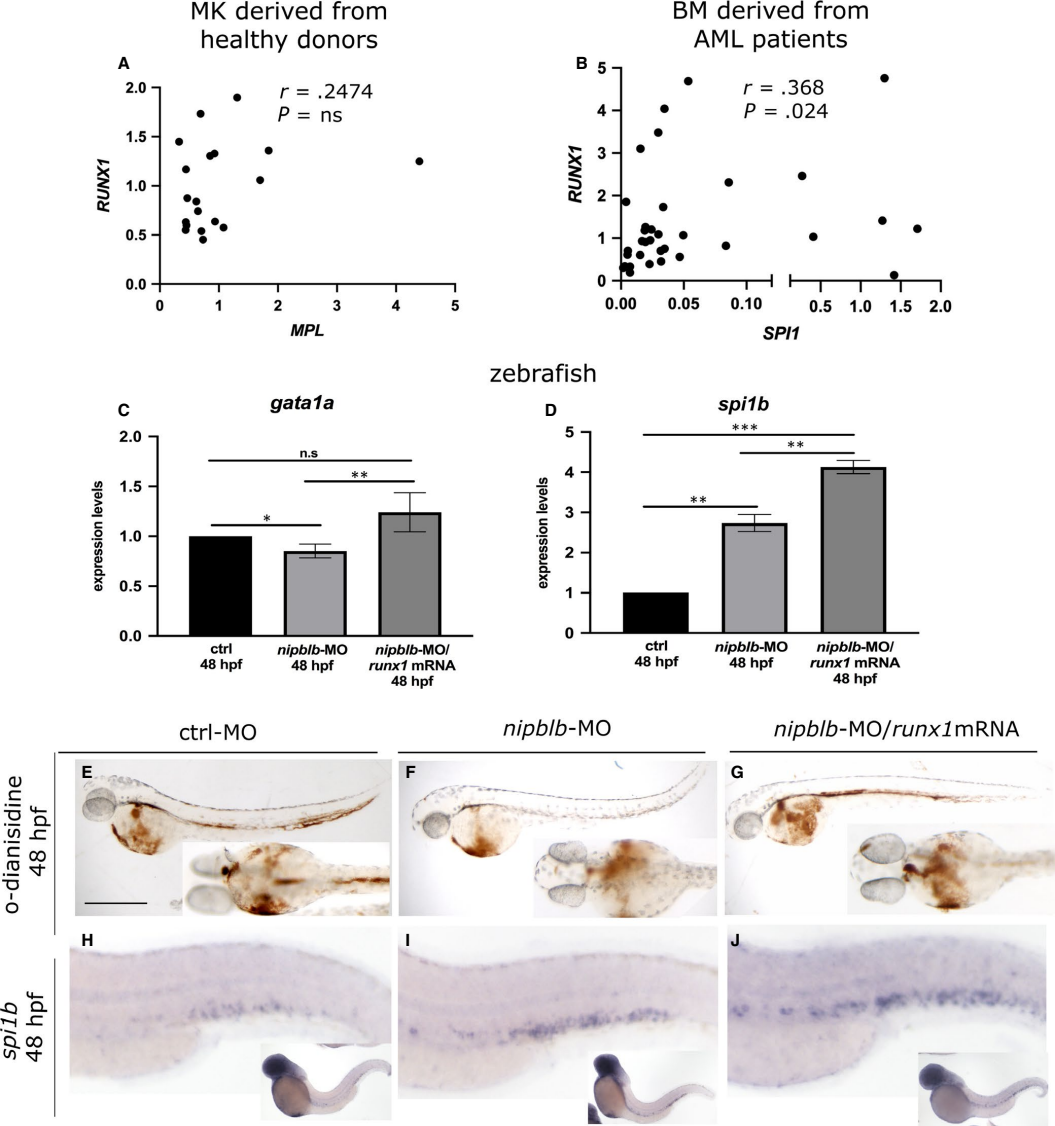
NIPBL‐mediated RUNX1 down‐regulation leads to impaired expression of RUNX1 target genes in both human and zebrafish. A, Spearman's correlation between *RUNX1* and *MPL* in cord blood megakaryocytes (MK) derived from healthy donors. B, Spearman's correlation between *RUNX1* and *SPI1* in bone marrow cells (BM) derived from 34 adult AML patients without aberrant *RUNX1* alterations (mutations or translocations). *r* = Spearman's correlation coefficient. C and D, RT‐qPCR analyses of 48 hpf ctrl‐, *nipblb‐* and *nipblb‐*MO/*runx1*mRNA‐injected embryos. C, The expression of the erythroid marker *gata1a* was decreased following *nipblb‐*MO injection in comparison with controls and rescued in *nipblb*‐MO/*runx1*mRNA‐injected embryos. D, The expression of the myeloid marker *spi1b* was increased in both *nipblb‐*MO‐ and *nipblb*‐MO/*runx1*mRNA‐injected embryos in comparison with controls. E‐G, *O*‐dianisidine staining showed a reduction of mature circulating erythrocytes in *nipblb*‐MO‐injected embryos at 48 hpf in comparison with ctrl‐MO. Co‐injection with the full‐length *runx1* mRNA rescues the *o*‐dianisidine reduction. Lateral views anterior to the left (upper panels) and ventral views of the anterior region (lower panels). H‐J, WISH analyses showed an increased expression of *spi1b* in *nipblb*‐MO‐ and *nipblb*‐MO/*runx1*mRNA‐injected embryos in comparison with ctrl‐MO. Scale bars indicate 100 μm in (E‐G) and 200 in μm in (H‐J)*.* One‐way ANOVA with Bonferroni correction, ^***^P < .001 ^**^ *P* < .01, ^*^ *P* < .05, n.s: non‐significant

We further verified whether the NIPBL‐mediated RUNX1 reduction affects the expression of *RUNX1* haematopoietic downstream targets*.* In MK cells derived from healthy donors, we observed a positive correlation between the expression of *RUNX1* and that of *MPL* gene, the marker of megakaryocyte/platelet differentiation (Figure 3A).^39^ In BM cells derived from AML human patients, we showed a positive correlation between the expression of *RUNX1* and its targets *SPI1*, the marker of myeloid precursors (Figure 3B).^40^ The expression of *runx1* targets *gata1a* and *spi1b* was investigated also in zebrafish in *nipblb*‐MO‐injected embryos and controls at 48 hpf. The expression of *gata1a*, analysed by RT‐qPCR, was significantly decreased following *nipblb* down‐regulation as a result of *runx1* reduction. Indeed, the injection of the *runx1*mRNA in the *nipblb*‐MO‐injected embryos rescued the *gata1a* expression (Figure 3C). Conversely, the expression of *spi1b* was significantly increased in both *nipblb*‐MO‐ and *nipblb*‐MO/*runx1*mRNA‐injected embryos (Figure 3D). Consistent with the role of *runx1* in the positive regulation of the erythroid lineage, mature circulating erythrocytes, visualized by *o*‐dianisidine staining at 48 hpf, were drastically reduced in *nipblb*‐MO‐injected embryos (70%; N = 140), compared to controls (Figure 3E‐F). This phenotype is not caused by alterations in vascular tree development as shown in the *Tg(fli1a:EGFP)^y1^* embryos (Figure S2), or absence of blood flow (data not shown). The reduction of *o*‐dianisidine‐positive erythrocytes was rescued in the 75% of the *nipblb*‐MO‐*runx1*mRNA‐injected embryos (N = 113) (Figure 3G), confirming that the phenotype is dependent on *nipblb*‐mediated *runx1* reduction.

WISH analyses of *spi1b* expression showed the increase of the transcript in the CHT of *nipblb*‐MO‐injected embryos (Figure 3H‐I) confirming the RT‐qPCR data and our previous findings.^34^ In agreement with the positive regulation exerted by *runx1* on *spi1b*, the injection of the *runx1*/mRNA further enhanced this phenotype (Figure 3J).^40^

**FIGURE 4:**
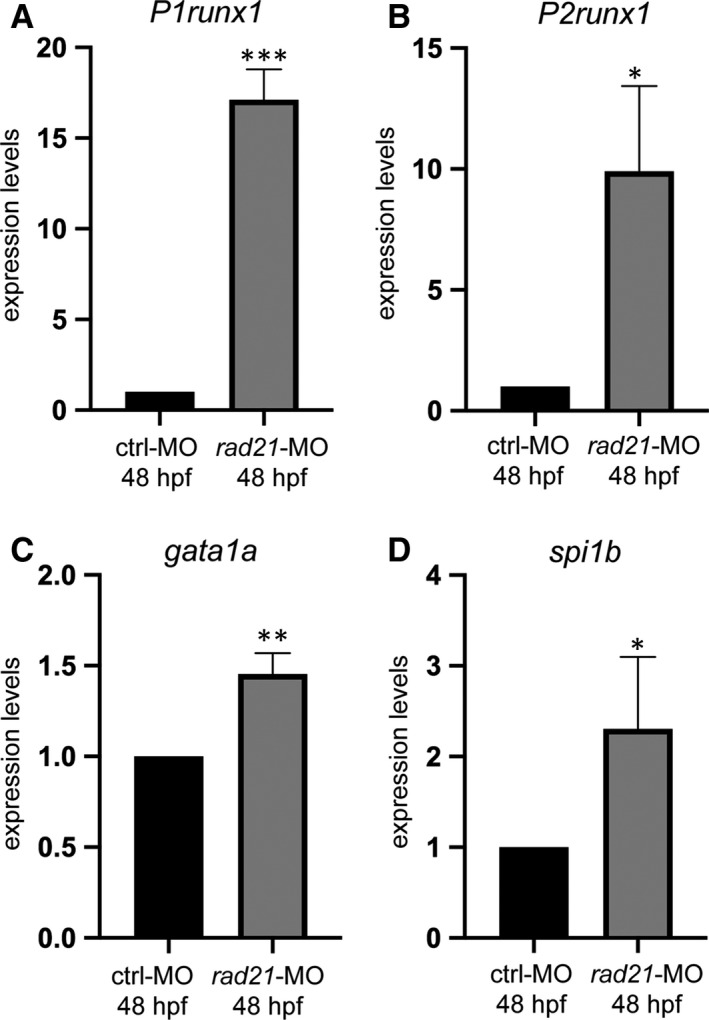
The down‐regulation of *rad21* in zebrafish enhances the expression of *runx1* and its downstream targets *gata1a* and *spi1b*. A‐D, RT‐qPCR analyses of 48 hpf ctrl‐ and *rad21‐*MO‐injected embryos. The expression of both *P1* (A) and *P2runx1* (B) isoforms and of *runx1* targets *gata1a* (C) and *spi1b* (D) was increased following *rad21‐*MO injection in comparison to  controls at 48 hpf*.* One‐way ANOVA with Bonferroni correction, ^***^ *P* < .001, ^**^P < .01, ^*^ *P* < .05, n.s: non‐significant

As it has been previously demonstrated that *rad21*, another member of the cohesin complex, regulates *runx1* in zebrafish embryos during primitive haematopoiesis,^25^, ^26^ we further verified the expression of *runx1* during definitive haematopoiesis following *rad21* down‐regulation by means of morpholino injection.^25^ *rad21*‐MO‐injected embryos at 48 hpf showed an increased expression of both *P1* and *P2runx1* isoforms and a consequent increase in the expression of the *runx1* downstream targets *gata1a* and *spi1b* (Figure 4A‐D). These data are in agreement with the negative regulation exerted by *RAD21* on *RUNX1* expression reported in the myeloid HL60 cell line.^26^

## 4 DISCUSSION

The transcription factor *RUNX1* is a pivotal gene in the development and differentiation of HSCs: as transcription factor, it controls the expression of master genes involved in megakaryocytes and myeloid lineages differentiation*,* and it interacts with different proteins fundamental during haematopoiesis. Somatic translocations and mutations of *RUNX1* are causative of haematological diseases such as myelodysplastic syndrome, acute myeloid leukaemia, acute lymphoblastic leukaemia, chronic myelomonocytic leukaemia and acute megakaryoblastic leukaemia with familial platelet disorder. In addition, dysregulation of *RUNX1* expression might lead to impaired haematopoiesis and the insurgence of a pathological condition. Among the genes discovered to regulate *RUNX1*, there is *RAD21*, a member of the cohesin complex, and the *CTCF* insulator.^26^ In human K562 cells and murine and zebrafish models, RAD21 and CTCF bind to a cis‐regulatory element (CRE) enhancer located in an intron between the *P1* and *P2RUNX1* promoters, associated with RNApolII.^41^ As cohesins preferentially bind to transcriptionally active genes and recruit RNAPolII and chromatin modifiers to activate gene transcription,^42^ it would have been expected that RAD21 positively regulates *RUNX1* transcription by binding to the CRE between *P1* and *P2* promoters. This finding is reported by Supernat and colleagues ^43^ in patients with endometrial cancers. However, in a zebrafish mutant for Rad21 the expression of *runx1* was reduced ^25^ and the *P1* and *P2runx1* isoforms were differently expressed: *P1* isoform was increased, while *P2* was not varied or even decreased following Rad21 depletion.^26^ Moreover, the silencing of *RAD21* in the human HL60 leukaemic cell line leads to an enhanced expression of *RUNX1* indicating that RAD21 might also repress *RUNX1* expression.^26^ In our study, we did not observe a significant correlation between the expression of *RAD21* and *RUNX1* neither in megakaryocytes derived from healthy donors, nor in bone marrow cells derived from a selected cohort of adult AML patients. However, we showed that during definitive haematopoiesis, the down‐regulation of *rad21* in zebrafish enhances the expression of both *P1* and *P2runx1* isoforms leading to impaired expression of the *runx1* downstream targets *gata1a* and *spi1b*.

The different members of the cohesin complex can exert similar or individual functions in the regulation of gene expression. For example, Zuin et al^44^ demonstrated that *NIPBL* binds to chromatin independently in time and space than other cohesins, revealing a new role for NIPBL as transcriptional regulator not linked to the cohesin complex. In this work, we demonstrate that NIPBL exerts a different regulation on *RUNX1* expression than RAD21. Indeed, in three different contexts: normal megakaryocytes derived from healthy donors, bone marrow cells derived from adult AML patients and zebrafish embryos with *nipblb* down‐regulation, we demonstrate a positive correlation between *NIPBL* and *RUNX1* expression.

The NIPBL‐mediated RUNX1 dysregulation affects the RUNX1 downstream targets responsible for the differentiation of the erythroid and myeloid lineages. *RUNX1* augmented GATA1‐mediated promoter activation; in this regard, the decrease in *RUNX1* transcription/activity leads to down‐regulation of the erythroid *GATA1* transcription factor.^45^ Interestingly, cohesins‐haploinsufficient cells presented enriched or depleted *GATA1* consensus binding sites indicating that they can modulate *GATA1* activity directly or through other molecules.^12^, ^46^

Also the *SPI* expression is positively regulated by RUNX1, facilitating the interaction between the *SPI* enhancer and its proximal promoter.^47^ Indeed, we observed a positive correlation between *RUNX1* and *SPI1* in human samples and in zebrafish when we forced *runx1* expression. However, following *nipblb* down‐regulation, we also observed an increase in *spi1b* expression according to our previous data.^34^ This result does not correlate with the *runx1* reduction and its positive activity on *spi1b* expression and raises three possibilities: first that the increased number of myeloid precursors, previously reported in zebrafish following *nipblb*‐MO injection,^34^ leads to an augmented number of cells expressing *spi1b* with a consequent total increase of *spi1b* transcript. Second, it has been reported that the chromatin structure at the *spi1b*/PU.1 locus could be differentially regulated during the different stages of haematopoiesis,^11^ suggesting the possibility that other mechanisms than RUNX1 might control *spi1b* expression. For example, we demonstrated that the canonical Wnt pathway, modulated by *nipblb*, has a pivotal role in regulating *spi1b* myeloid expression during definitive haematopoiesis in zebrafish.^34^ Moreover, in vitro and in vivo studies demonstrated that forced expression of *gata1* down‐regulates *spi1b*, while forced expression of *spi1b* down‐regulates *gata1*.^48^, ^49^, ^50^ In this scenario, the *nipblb*‐mediated *runx1* down‐regulation might lead to *spi1b* enforced expression that, in turn, reduces *gata1a* expression. Alternatively, the two *P1* and *P2runx1* isoforms might exert different functions on *spi1b* regulation. Indeed, as for the case of Rad21 zebrafish mutants,^26^ we demonstrated that the down‐regulation of *nipblb* differently affects the two isoforms by significantly reducing only the *P2runx1*. Third, it has been demonstrated that NIPBL might regulate SPI1 by itself, encompassing the Runx1 regulation.^44^

Although in this work we did not address the mechanism through which NIPBL regulates *RUNX1* expression, we demonstrated that NIPBL positively regulates *RUNX1* transcription and that the link between *NIPBL* dysregulation and *RUNX1*‐driven haematopoietic defects might  explain haematological malignancy occurrence. Thus, also in the absence of *RUNX1* translocation or mutations, additional factors such as defects in the expression of *NIPBL* observed in AML patients might contribute to haematological diseases.

## CONFLICT OF INTEREST

The authors declare no competing financial interest.

## AUTHOR CONTRIBUTION

MM, GF, CS, AR, AP, MCP, GG and LF contributed to the study. MP and MF provided patients samples. EB, AP, GC, AB, and AM designed and performed the experiments and analysed the data. AP designed and organized the experiments and analysed the data; and wrote the manuscript.

## Supporting information

Fig S1‐S2

## Data Availability

The data that support the findings of this study are available from the corresponding author upon reasonable request.
