# Supplementary material for: Dysregulation of NIPBL leads to impaired RUNX1 expression and haematopoietic defects
Source: J Cell Mol Med. 2020 Apr 23;24(11):6272–82. doi: 10.1111/jcmm.15269 (PMC7294146; doi:10.1111/jcmm.15269)
Supplement: Supplementary file 1 — Fig S1‐S2 [file JCMM-24-6272-s001.docx]

**Supplementary material**

**
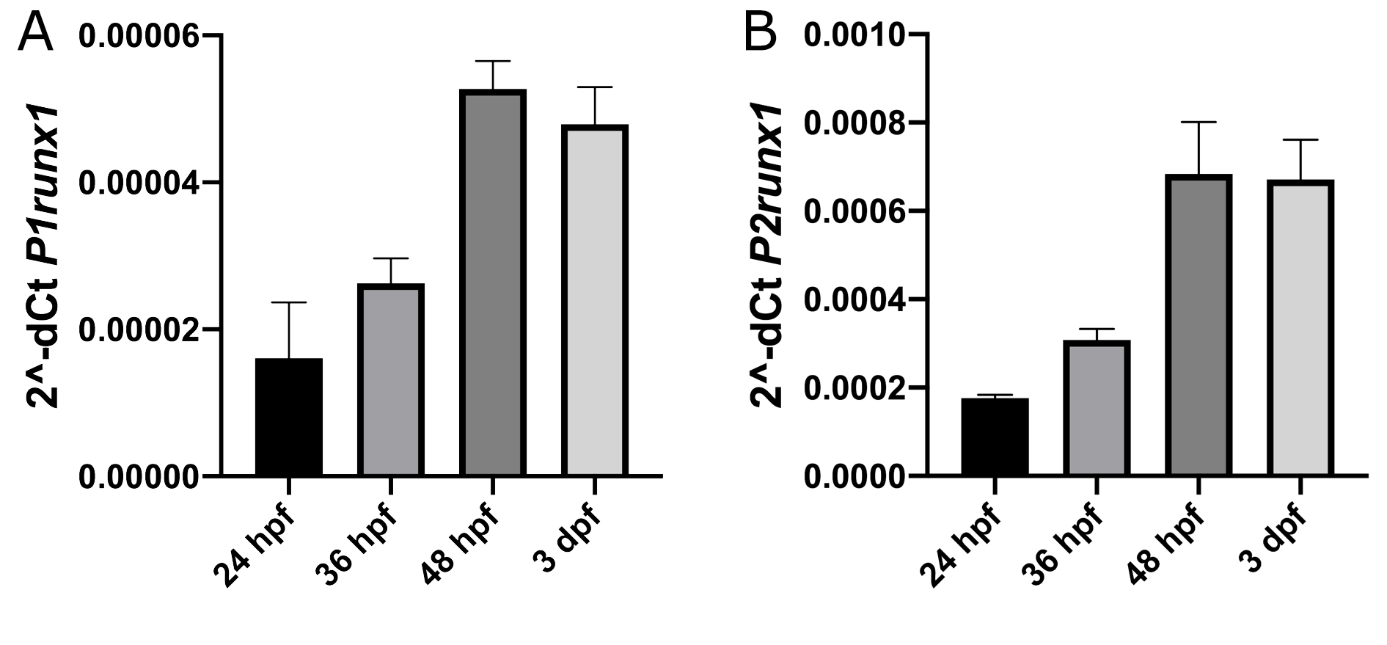
Suppl. Fig. S1 Expression analyses of *P1* and *P2runx1* isoforms at different stages of zebrafish development.** *(A-B)* RT-qPCR analyses of *P1* *(A)* and *P2runx* *(B)* isoforms at different developmental stage of zebrafish: somitogenesis, 24, 36, 48 hpf and 3 dpf.

**
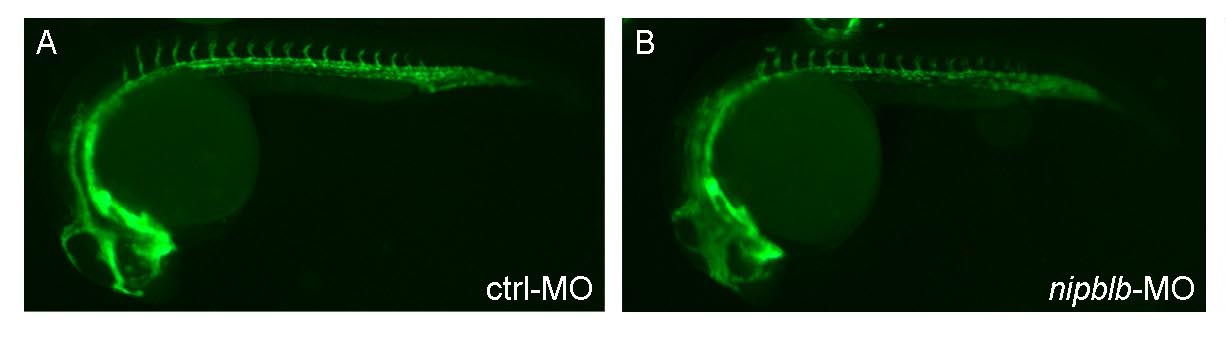
**

**Suppl. Fig. S2 Vessel formation is not affected following *nipblb*-MO in *Tg(fli1a:EGFP)^y1^* embryos.** *(A-C)* Fluorescent images of *Tg(fli1a:EGFP)^y1^* controls *(A)* and *nipblb*-MO *(B)* injected embryos showed a normal pattern of vessel formation. Scale bar: 100 μm.
